# Does Tetralogy of Fallot affect brain aging? A proof-of-concept study

**DOI:** 10.1371/journal.pone.0202496

**Published:** 2018-08-21

**Authors:** Marina Codari, Giacomo Davide Edoardo Papini, Luca Melazzini, Francesca Romana Pluchinotta, Francesco Secchi, Mario Carminati, Alessandro Frigiola, Massimo Chessa, Francesco Sardanelli

**Affiliations:** 1 Unit of Radiology, IRCCS Policlinico San Donato, San Donato Milanese, Milan, Italy; 2 Department of Biomedical Sciences for Health, Università degli Studi di Milano, San Donato Milanese, Milan, Italy; 3 Unit of Pediatric and Adult Congenital Heart Disease, IRCCS Policlinico San Donato, San Donato Milanese, Milan, Italy; 4 Unit of Pediatric and Adult Cardiac Surgery, IRCCS Policlinico San Donato, San Donato Milanese, Milan, Italy; Kurume University School of Medicine, JAPAN

## Abstract

The impact of congenital heart disease on brain aging has not been extensively investigated. We evaluated cerebral microbleeds and white matter hyperintensities on brain magnetic resonance imaging in adult patients with tetralogy of Fallot (ToF). Ten ToF patients (6 women, 4 men; aged 21–58 years; New York Heart Association [NYHA] class 1–2) were prospectively enrolled and underwent a T1-weighted, a T2-weighted dark fluid, and a T2*-weighted scans. Ten age- and sex-matched controls were prospectively recruited and subjected to the same acquisition protocol. Cerebral microbleeds (CMBs) were manually counted while white matter hyperintensities (WMHs) were segmented using ITK-Snap. Wilcoxon signed-rank test, Spearman correlation, and Bland-Altman statistics were used. The median (interquartile range [IQR]) age was 45.0 (30.5–49.5) years in ToF patients and 46.0 (30.5–49.8) years in controls. The median (IQR) of the number of CMBs was 6.0 (4.0–7.8) in ToF patients and 0 (0.0–0.0) in controls (*p* = 0.002). The WMHs burden was 2,506 (1,557–2,900) mm^3^ for ToF patients and 2,212 (1,860–2,586) mm^3^ for controls (*p* = 0.160). Moreover, a positive significant correlation was found between the WMHs burden and the NYHA class (ρ = 0.80, *p* = 0.005). Inter-operator concordance rate for the presence/absence of CMBs was 90%; the reproducibility for the WMHs burden was 77%. In conclusion, we found more cerebral microbleeds and a higher WMHs burden in adult ToF patients than in controls. This preliminary comparison supports the hypothesis of an early brain aging in ToF patients. Larger studies are warranted.

## Introduction

Thanks to modern advancements in cardiac surgery, the adult population living with complex congenital heart disease (CHD) has increased over the last decades. In particular, the survival of those affected by the most common cyanotic CHD, namely Tetralogy of Fallot (ToF), has been documented up to the 7^th^ or 8^th^ decade of life [1].

Clinicians are facing the challenge of an aged ToF population, including the possibility of adverse outcomes even many years after cardiac surgery [1]. In this scenario, monitoring both the cardiovascular and the central nervous system of adult ToF patients has become a major issue. Even though prevalence for traditional cardiovascular risk factors and outcomes were already investigated in adult ToF population [2], at present no reports have ever investigated the impact of complex CHD on brain aging.

The aging process entails several brain changes over time. Cerebral microbleeds (CMBs) and white matter hyperintensities (WMHs) are neuroimaging biomarkers of cerebral small vessels disease (cSVD), typically investigated through magnetic resonance imaging (MRI) [3]. CMBs, presenting as small black dots on T2*-weighted gradient-echo images, have a prevalence of about 25% in the elderly and tend to increase with age [4]. In addition to being imaging markers of cSVD and biological aging [5], CMBs were found to be independent predictor of increased risk of stroke, dementia, and all-cause mortality [6]. Together with CMBs, WMHs, seen on MRI using T2-weighted dark-fluid images, are considered a valid biomarker of brain aging, also in asymptomatic subjects [7]. They are associated with an increased risk of stroke, cerebral atrophy, cognitive impairment, and transition to disability [8–10].

We hypothesized that ToF patients could exhibit signs of brain aging, construed as an increased cSVD burden, earlier than healthy peers. This hypothesis was driven by the consideration of an earlier onset of cardiovascular risk factors for brain injuries such as, among others, hypertension, metabolism disorders and dysrhythmias, impacting the brain and contributing to a precocious neurocognitive decline in these patients [11].

In support of this argument, an increased CMBs count was observed in pediatric CHD patients after cardiac surgery [12], while a high prevalence of WMHs, qualitatively [13,14] and semi-quantitatively [15] assessed on MRI images, has been reported in adult patients with unrepaired CHDs.

The investigation of the general linkage between cardiovascular and brain health is gaining importance in order to develop tailored strategies to prevent or delay cognitive impairment in patients affected with cardiovascular disease [11,16]. Thus, brain MRI studies in adult CHD patients can offer insights into this key relation. In this light, this proof-of-concept study focused on the assessment of CMBs and WMHs, herein used as hallmarks of age-related brain changes, in adult ToF patients versus a control group.

## Patients and methods

### Dataset

We recruited 10 patients: 8 subjects affected by ToF and 2 patients with similar clinical conditions (pulmonary valve atresia combined with interventricular septal defect) who came to our institution to undergo a scheduled cardiac MRI from June 2017 to May 2018. In parallel, 10 age- and sex-matched control subjects were asked to participate to this study. The ethical committee of the San Raffaele Research Hospital approved this study on 8th June 2017 (protocol name: LEUCO) and written informed consent was obtained from all patients. All subjects underwent brain MRI at our institution from June 2017 to May 2018.

Exclusion criteria for both patients and controls were: age < 18 years; inflammatory, infectious, demyelinating or dysmyelinating diseases of the central nervous system; ischemic, haemorrhagic, or traumatic brain events with possible gliotic, malacic, or lacunar sequelae; mendelian or mitochondrial genetic diseases of the central nervous system, including cerebral autosomal dominant arteriopathy with subcortical infarcts and leukoencephalopathy; cerebral amyloid angiopathy; cerebral arteriovenous malformations; primary or metastatic brain neoplasms; previous cranial/brain surgery; patent oval foramen; pregnancy; migraine with aura [17].

Controls were matched to patients for age and sex. One year age absolute difference between ToF and controls was deemed acceptable

In addition to imaging data, we collected information about patients’ clinical condition and surgical history, such as: number of surgical interventions with extracorporeal circulation (ECC), age at corrective surgery (expressed in months) and New York Heart Association (NYHA) class.

### MRI protocol

**Fig 1:**
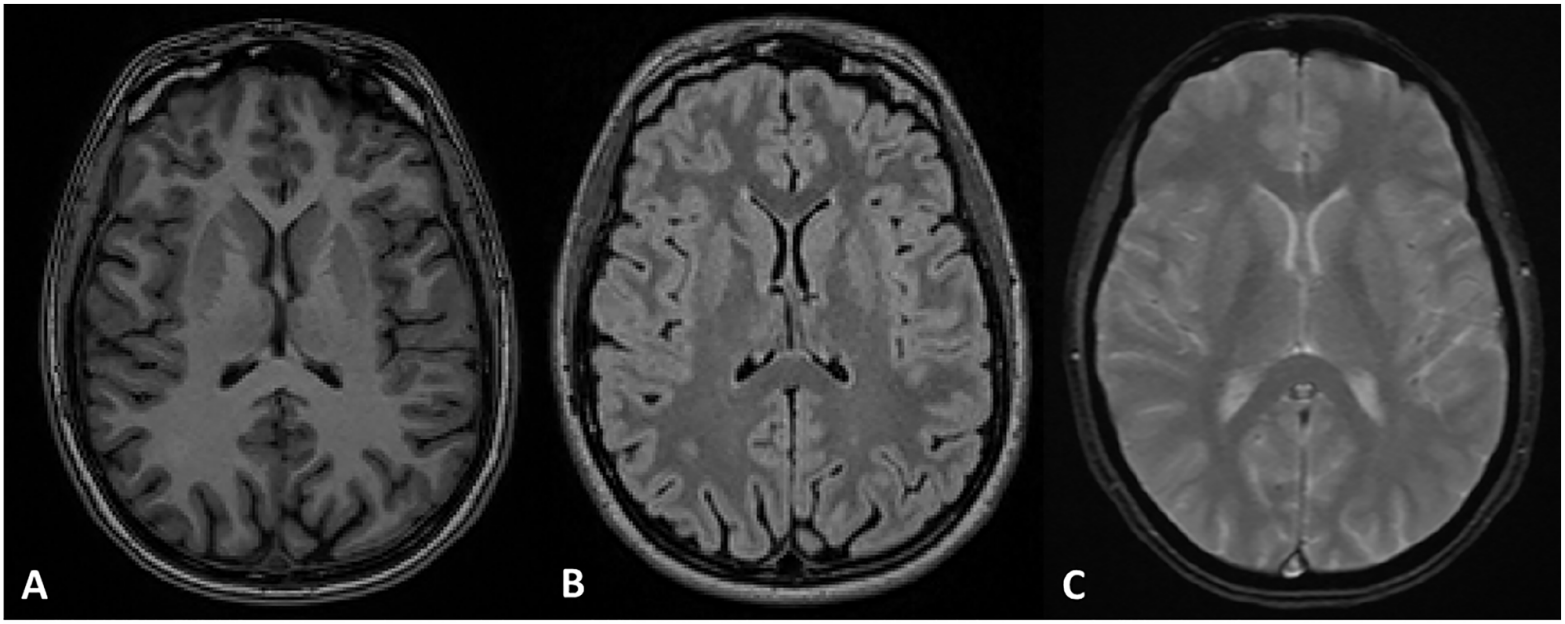
Examples of images obtained from the MRI protocol. A) Three-dimensional T1-weighted image; B) Three-dimensional dark-fluid T2-weighted image; C) two-dimensional axial T2*-weighted image.

The MRI protocol included the following sequences: a three-dimensional T1-weighted scan; a three-dimensional T2-weighted dark-fluid scan; and a two-dimensional axial T2*-weighted scan (Fig 1).

Examinations were performed using a 1.5-T scanner (Magnetom Aera, Siemens Healthineers, Erlangen, Germany). Sagittal three-dimensional T1-weighted images were obtained using a magnetization-prepared rapid gradient-echo sequence with the following technical parameters: inversion time [TI] 900 ms; echo time [TE]: 2.26 ms; repetition time [TR]:2,200 ms; flip angle [FA]: 8°; resolution 0.98×0.98×1.00 mm^3^). Sagittal three-dimensional dark-fluid T2-weighted images were obtained using a “sampling perfection with application optimized contrasts using different flip angle evolution” (SPACE) sequence (TI 1,800 ms; TE 335 ms; TR 5,000 ms; FA 120°; resolution 1.02×1.02×1.00 mm^3^). Axial T2*-weighted images were obtained using a spoiled gradient-echo sequence (TE 25 ms; TR 965 ms; FA 20°; resolution 0.45×0.45×4.00 mm^3^;). brain MRI scans were acquired in controls using a second 1.5 T scanner (Magnetom Symphony Tim, Siemens Healthineers, Erlangen, Germany) using a similar sequence protocol: T1-weighted scan (TI 900 ms; TE 2.8 ms; TR 2,200 ms; FA 8°; resolution 0.98×0.98×1.00 mm^3^); dark-fluid T2-weighted scan (TI 2,200 ms; TE: 358 ms; TR: 6,000 ms; FA 120°; resolution 1.02×1.02×1.00 mm^3^): 2D T2*-weighted scan (TE 26 ms; TR 822 ms; FA 20°; resolution 0.45×0.45×4.00 mm^3^)

### Image interpretation and biomarker quantification

The image reading was performed by a PhD student specifically trained by a neuroradiologist with 6 years of clinical experience. CMBs were counted on T2*-weighted images. To quantify the WMHs burden, a semi-automated segmentation method based on local intensity thresholds was used and then manually refined. ITK-Snap software was used to this aim [18]. Finally, the total burden of the WMHs was expressed as their volume extent in mm^3^.

To assess and inter-operator agreement, the segmentation was performed by a second reader, a medical student previously trained by a neuroradiologist with 6 years of clinical experience in a subsample of subjects (5 randomly selected patients and their respective controls). As for WMH, to evaluate inter-operator agreement CMBs were counted by two operators in the same subsample of subjects.

### Statistical analysis

Kolmogorov–Smirnov test was applied to check normality of data distribution. The Wilcoxon signed-rank test was then applied to compare the two groups. Moreover, the non-parametric Spearman’s correlation coefficient (ρ) was used to quantify the association between MRI biomarkers (i.e. WMHs burden and CMBs count) and demographics or clinical/surgical data. Finally, we investigated and inter-observer variability using the Bland-Altman analysis for the WMHs burden and the percentage of concordance between operators in CMBs counting. Statistical significance level was set at *p*-value ≤ 0.05. Statistical analysis was performed using Matlab r2016a (MathWorks, Natick, MA, USA).

## Results

The patient group consisted of 10 subjects (6 males and 4 females), with a median age of 45.0 years (interquartile range [IQR] 30.5–49.5 years; range 22–64 years). The control group was composed of age-and sex-matched prospectively selected controls, with a median age of 46.0 years (IQR 30.5–49.8 years; overall range 22–63 years). Table 1 summarizes clinical and surgical history data of our patient sample.

**Table 1:** Summary of patients’ clinical and surgical history. Age, sex, number and type of undergone cardiac surgical procedures in the analysed sample.

	Age [years]	Sex	NYHA class	Age at corrective surgery [months]	Cardiac surgery using ECC	PPVI	Total procedures
Patient 1	52	M	2	48	4	-	3
Patient 2	30	F	2	16	1	2	3
Patient 3	32	M	1	6	2	1	3
Patient 4	48	M	2	108	1	1	2
Patient 5	47	F	2	228	1	-	1
Patient 6	22	M	2	11	2	1	3
Patient 7	28	M	1	12	2	-	2
Patient 8	50	F	1	96	1	-	1
Patient 9	64	M	2	48	2	-	2
Patient 10	43	F	2	36	2	1	3

Age, sex (M = male, F = female), number and type of undergone cardiac surgical procedures in the analysed sample. NYHA = New York Heart Association; ECC = extracorporeal circulation; PPVI = percutaneous pulmonary valve implantation; CMBs = cerebral microbleeds; WMHs = white matter hyperintensities.

**Fig 2:**
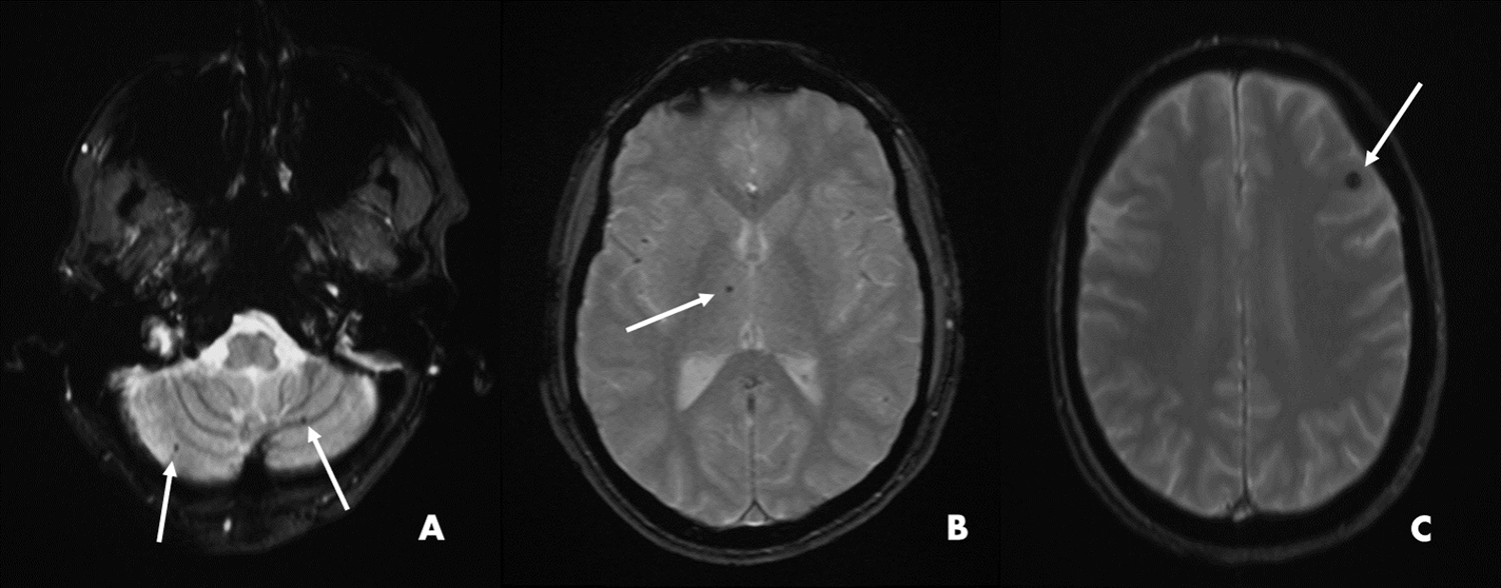
Cerebral microbleeds. White arrows highlight cerebral microbleeds (CMBs) on T2*-weighted scans depicted according to brain location in three patients affected by Tetralogy of Fallot. (A) Two cerebellar CMBs in a 50-year-old female. (B) Right thalamic CMB in a 22-year-old male. (C) Left frontal CMB in a 43-year-old female.

WMHs were identified in 100% of our samples, while CMBs were identified in 100% of ToF subjects and in one control only. Due to the non-normal distribution of WMHs volume and CMBs count in both patient and controls (*p*<0.001), nonparametric tests were used. Median and IQR value of CMBs count were 6.0 (4.0–7.8) in ToF patients and 0.0 (0.0–0.0) in controls (*p* = 0.002). Fig 2 shows several examples of CMBs visible on T2*-weighted MRI of three ToF patients.

**Fig 3:**
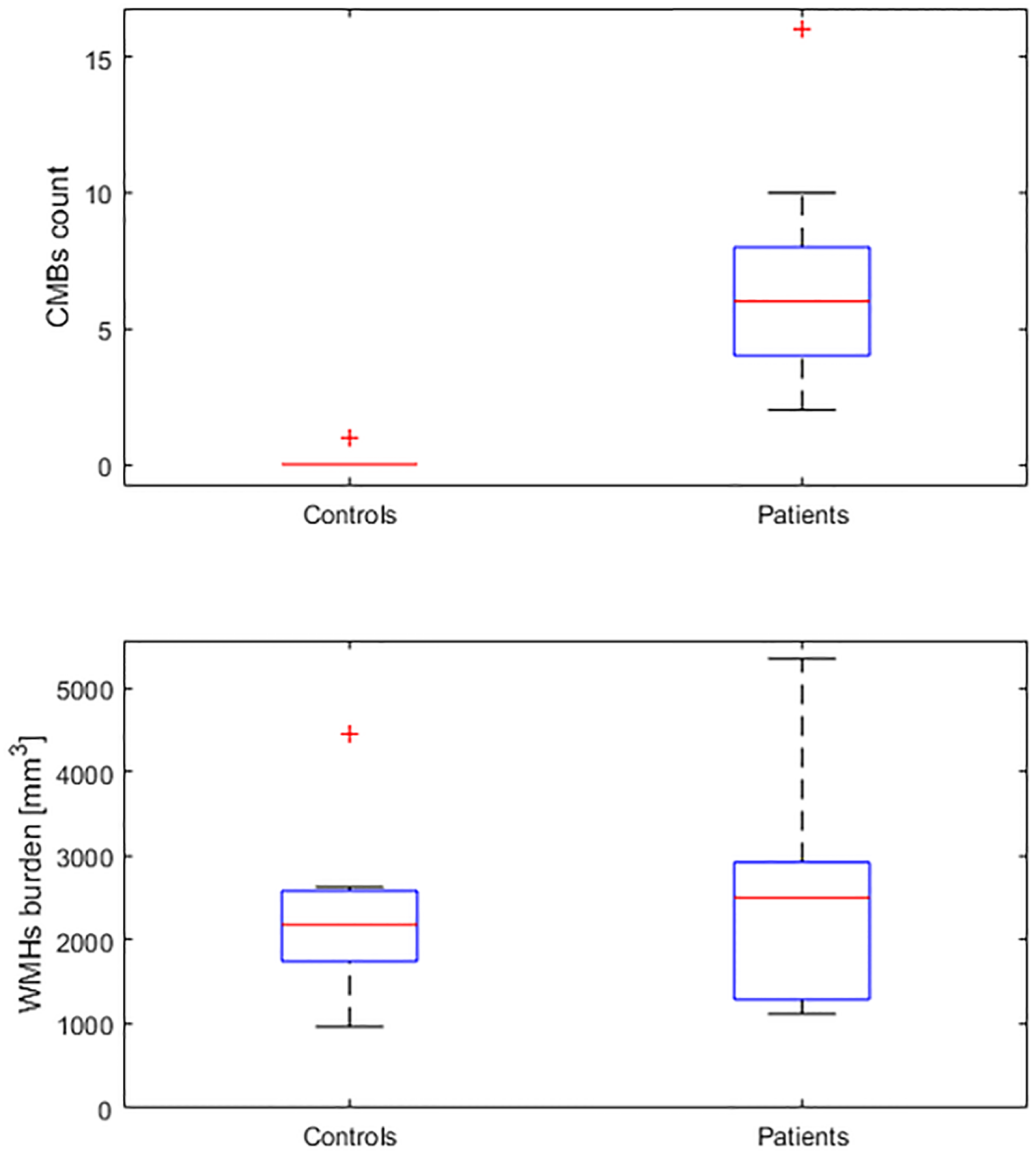
Box-plot data depiction. Box-plot of the estimated white matter hyperintensities (WMHs) burden and cerebral microbleeds (CMBs) count in patients with Tetralogy of Fallot and in control subjects.

Median (IQR) of the WMHs burden was 2,506 (1,557–2,900) mm^3^ in ToF patients and 2,184 (1,774.5–2542.8) mm^3^ in controls (*p* = 0.160). Fig 3 shows the distribution of WMHs burden and CMBs count in patient and controls.

**Fig 4:**
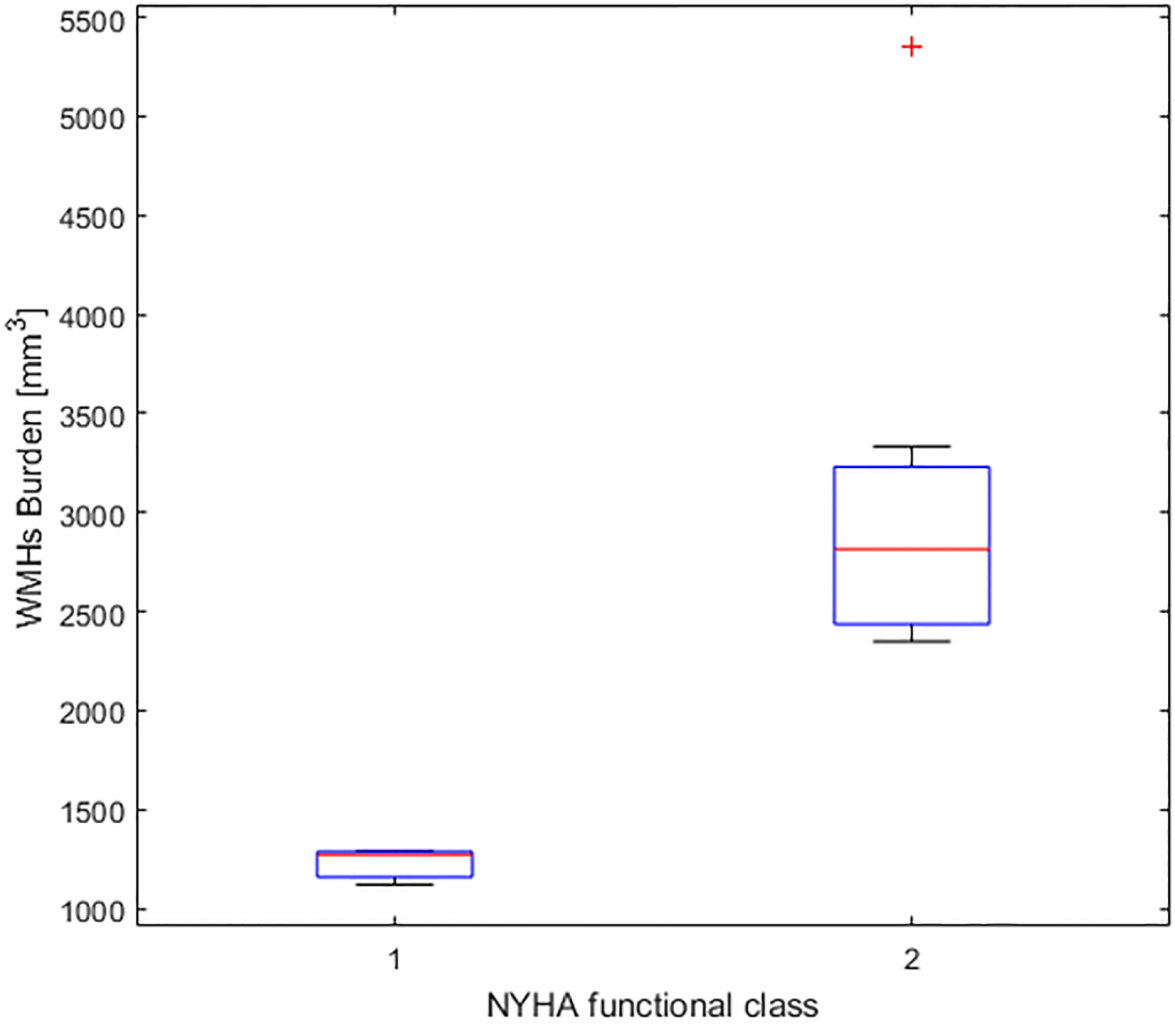
White matter hyperintensities (WMHs) burden and cardiac function. Boxplot representation of WMHs volume in ToF subjects graded I or II class of the New York Heart Association (NYHA) scale.

**Fig 5:**
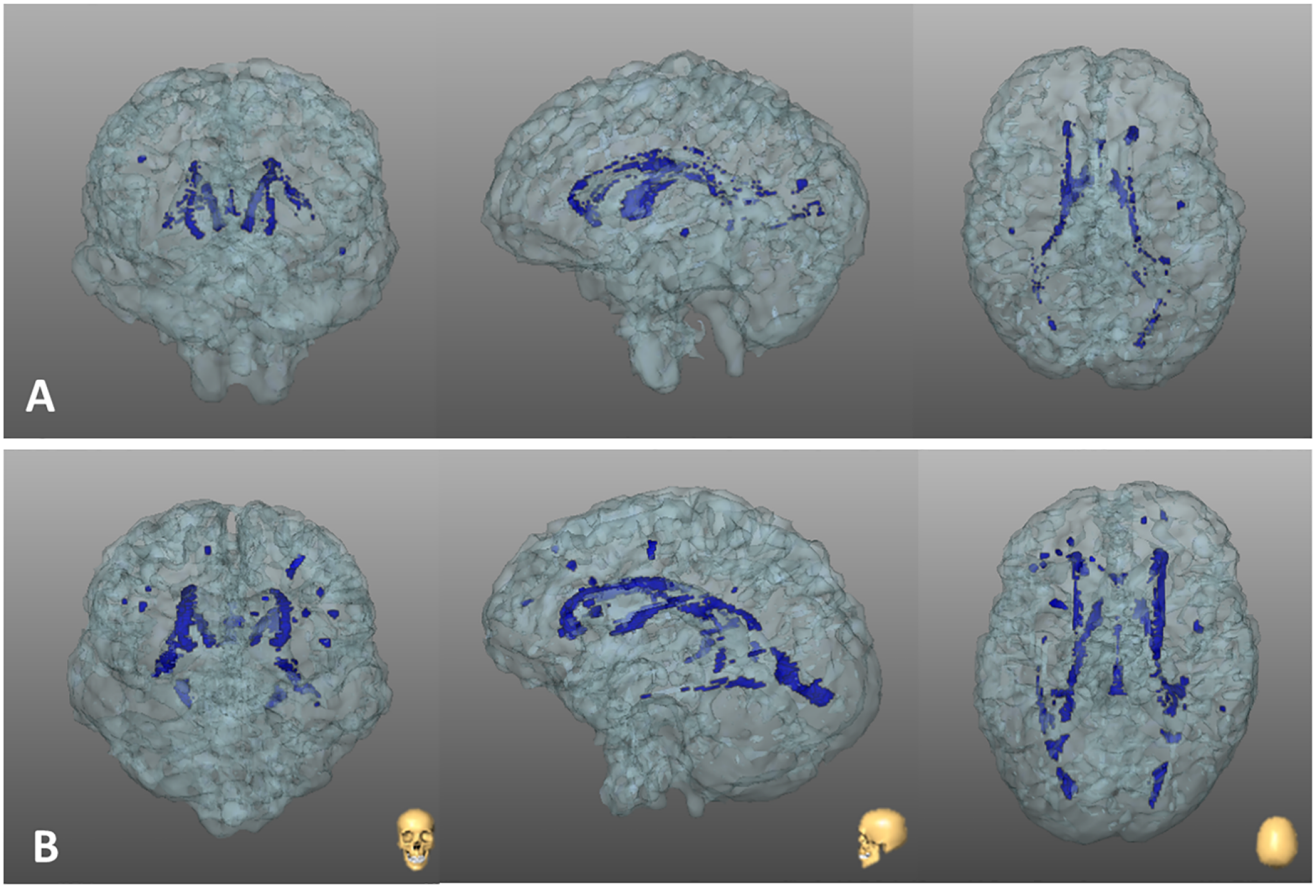
Volumetric rendering of total brain volume in two subjects with Tetralogy of Fallot. A) NYHA I, 50 years-old female. B) NYHA II, 52 years-old male. White matter hyperintensities are labeled in blue.

A positive, albeit not significant, correlation between WMHs burden and age was found in both controls (ρ = 0.49; *p* = 0.154) and patients (ρ = 0.190; *p* = 0.608). The same trend was observed when assessing the association between CMBs and age in both patient (ρ = 0.12; *p* = 0.737) and controls (ρ = 0.41; *p* = 0.244). Spearman’s rank correlations among MRI biomarkers of cSVD are reported in Table 2. The only significant association was that between NYHA class and WMHs burden in patients (*p* = 0.006). Fig 4 shows the distribution of WMHs burden in subject belonging to different NYHA classes. Fig 5 shows the volumetric representation of segmented WMHs in two patients of comparable age graded with different NYHA scores (I and II).

**Table 2:** MRI biomarkers and clinical data. Spearman’s rank correlations between MRI biomarker of cerebral small vessel disease and clinical/surgical data.

MRI biomarkers	Clinical data	ρ	p-value
**WMHs**	**NYHA class**	0.798	0.006
	**Age at corrective surgery**	0.128	0.725
	**Surgical interventions with ECC**	0.281	0.431
	**Total procedures**	0.178	0.623
**CMBs**	**NYHA class**	-0.268	0.455
	**Age at corrective surgery**	-0.107	0.769
	**Surgical interventions with ECC**	0.384	0.273
	**Total procedures**	0.106	0.771

M = male; F = female; NYHA = New York Heart Association; ECC = extracorporeal circulation; PPVI = percutaneous pulmonary valve implantation; CMBs = cerebral microbleeds; WMHs = white matter hyperintensities.

Inter-operator Bland-Altman analysis for reproducibility in quantifying WMHs burden showed a bias of 196.7 mm^3^, a coefficient of repeatability of 530.1 mm^3^, an average measurement of 2,226.4 mm^3^, resulting in a reproducibility equal to 77%. On the other hand, inter-operator concordance rate among readers in CMBs count resulted equal to 60%. When expressed on a dichotomous scale (present/absent) the concordance rate in CMBs detection was equal to 90%. Detailed information on the database used in this study are listed in S1 File.

## Discussion

To proof the concept of a possible earlier brain aging in CHD patients, we compared the cSVD burden in a small group of adult ToF patients with that in a sample of age- and sex-matched controls. We worked under the well-established assumption that CMBs on T2*-weighted images and WMHs on dark-fluid T2-weighted images represent a biomarker of brain aging [3], herein investigated in the most common cyanotic CHD [1].

It is acknowledged that CHD newborns show signs of white matter vulnerability, possibly due to abnormal brain development in utero [19] and perform lower scores in cognitive and muscle function scales than normative means as they reach childhood [20]. Nevertheless, the fact that impaired neurodevelopment becomes early brain aging and neurocognitive decline in adult CHD patients has not yet been proven [11].

In this study, CMBs were found to be significantly higher in patients, with controls basically not displaying any of them (*p* = 0.002). The detection of CMBs on GRE images represents a challenge. Radiologists may slightly disagree when estimating their size or burden, like in our case (concordance rate of 60%). However, when CMBs were dichotomically described only as present or absent, the concordance rate between the two readers became excellent (90%). In this scenario, due to the overwhelming absence of CMBs in control sample, readers’ discordance can be considered as a marginal issue.

The presence of CMBs could be related to disease severity, number and type of therapeutic procedures, the precocious occurrence of traditional cardiovascular risk factors and their interplays. In fact, the outcome in terms of brain aging could be ostensibly secondary to a mutually self-sustaining circle among the above mentioned contributory causes.

Cardiac surgery may harm the brain through cerebral micro- o macro-embolism and hypoperfusion, the latter being worsened by the inflammatory events resulting from cardiopulmonary bypass and ischemia/reperfusion injury [21]. Moreover, percutaneous valve-replacement manoeuvres that call for valve manipulation, balloon expansion, and big vessels mechanical stress could generate small cerebral embolic infarcts [22]. Even though post-operative lesions represent acute injures rather than a long-term outcome from small vessel diseases under consideration, still their chronic MRI correlates may be alike.

Differently form CMBs, WMHs burden in ToF patients was not significantly higher than in controls (*p* = 0.160). We did not resort to visual rating scale due to their well-established lack of reliability in comparison to volumetric assessments, particularly in low-burden WMHs, which represent the vast majority of cases [23]. In this study, we quantified WMHs burden with a semi-automatic method improved with manual contouring, which showed an acceptable intra-operator reproducibility. Considerable variability was encountered in patients, whose WMHs volumetric burden was even very low or very high, with controls displaying a more homogeneous trend. It is reasonable to expect that this not significant difference would become more pronounced in further studies endowed with larger samples.

Interestingly, among the associations we analysed, the one between NYHA class and WMHs burden was the only significant, coherently with previously reported results in cardiac failure patients [24]. The difference between ToFs and control subjects was remarkable (see Fig 4), thus supporting the concept of a strong association among cardiac function, brain status, and daily living physical activities. How cardiac function may affect white matter integrity can be visually rendered by the examples shown in Fig 5. We hypothesize that the inclusion of subject belonging the third and fourth NYHA classes will strength this association, thus spotlighting the need to further investigate the link between cardiac function and brain status in ToF subjects.

We found a trend for a positive correlation between all imaging biomarkers of cSVD and age in both groups, probably not significant due to the small sample size of this preliminary study. While varying degrees of cSVD were found in young individuals of both groups, a definite upward trend was found in the elderly. These findings are consistent with the well-known phenomenon of cSVD increase with aging of general population [3–5, 25].

Besides, the use of CMBs and WMHs as a neuroimaging biomarker of brain aging deserve a general comment. The underlining pathology for WMHs is an open issue. Arteriolosclerosis, subependymal gliosis, axonal damage, and demyelination at different stages were previously reported on brain autopsy studies [26, 27]. Thus, WMHs lack pathological specificity and are associated with a variety of both vascular and inflammatory conditions. Conversely, among MRI findings related to cSVD, CMBs are more specific. They represent a direct effect of microvascular leakiness, which causes blood breakdown products to extravasate through a damaged blood-brain barrier (BBB) and be engulfed by brain macrophages and microglia. Thus, the failure of the BBB as a prominent pathophysiological mechanism for CMBs occurrence may also play a role for other manifestations of cSVD that acknowledge a common ischemic genesis and are indeed strongly associated with CMBs, WMHs at first [28]. Hence, it is supposed that a disrupted BBB and an extravasation of cells and plasma components may lead to demyelination and gliosis, contributing to WMHs initiation or progression [29] as well as hemosiderin deposits occurrence, i.e. CMBs.

In our study, we hypothesize that the increased burden of cSVD in CHD patients, whose cardiovascular risk propensity is undoubtedly high, may be mediated by vascular damage, presumably via cyanosis prior to surgical repair, surgical brain damage and cardiac failure, eventually leading to BBB disruption and subsequent MRI-detectable changes. However, it is uncertain whether age at first corrective surgery may impact cSVD neuroimaging findings in adults, since postoperative lesions in children have been shown to regress short after surgery [30]. Indeed, our results did not show a significant correlation between age at repair surgery, namely time of cyanosis prior to surgical correction, and either WMHs burden (p = 0.725) or CMBs count (p = 0.769).

Occurrence of CMBs right after cardiac surgery has been previously reported, both in children with CHD [12] and in adults undergoing on-pump coronary artery bypass grafting [31]. It is however unclear whether those micro-haemorrhages observed on brain MRI may persist long after surgical repair [5]. That is to say, we cannot establish if the strikingly high CMBs burden we encountered was brought about by chronic microvascular damage or heart surgery. We believe they both may play a role in harming cerebral vessels so to contribute to brain aging and vulnerability. In support of this argument, we did not find any correlation between number of surgeries performed with ECC or number of total cardiac procedures and cSVD, so that cardiac surgery on its own cannot account for the CMBs burden we observed.

In the specific case of our sample, the joint presence of four CMBs in a 64-year-old patient, with a record of three cardiac surgeries (two of them requiring ECC), and a four-fold CMBs count in a 50-years-old patient who underwent a single surgical procedure (with ECC) highlights the weakness of the possible association between surgical history and number of CMBs.

Stated the above, residual issues concern the assessment of the imaging biomarkers under consideration. Spoiled GRE and other paramagnetic-sensitive MRI sequences allow to detect CMBs [6], although it may be difficult to tell them apart from their mimics, particularly when phase images are not employed [5]. To improve the current MRI protocol to overcome CMBs detection issues, we will introduce sequences for susceptibility-weighted imaging (SWI) aimed at visualizing them with the highest *in vivo* sensitivity [28].

Dark-fluid T2-weighted images can help to distinguish WMHs from silent brain infarcts that appear as CSF-filled cavities or lacunes. However, they can overestimate WMHs burden: almost a third of lacunar infarctions do not develop lacunes and so are indistinguishable from WMHs [32]. Importantly, WMHs represent only radiological finding, not a clinical finding. A blurred border exists between the “incomplete infarction” [33] at the bottom of white matter rarefaction and those silent strokes that withstand cavitation. Therefore, it is reasonable to consider WMHs and not-cavitating silent brain infarcts as a continuous entity for the purposes of this study.

This preliminary study has some limitations. First, the small sample size, which did not prevented us to obtain a statistical significance for the difference in CMBs occurrence in patients and controls. Conversely, the lack of statistical power limited the assessment of correlation between cSVD burden and age in both patients and controls, to be investigated in future larger studies. Second, in respect of our primary aim, median age and interquartile range of 45.0 (29.5–50.5) years for the patients’ group are lower than desirable. A confirmation and a strengthening of the observed results for elder individuals is thus needed.

Third, we did not investigate the possible clinical correlate of the increased cSVD burden in patients. The latter will be a necessary aim of larger transversal and longitudinal studies that shall be provided with neuropsychological assessments.

We will further investigate thoroughly on the topic of cSVD and brain aging on CHD patients. We will first implement our MRI protocol with new SWI sequences to evaluate non-heme iron deposition on basal ganglia, which have been proven to be a promising biomarker of neural and cognitive declines in normal aging [34].

We will also administer a questionnaire on lifestyle and psycho-social status specifically designed for adult CHD patients. Further clinical data such as blood pressure and pulse oximetry will be collected to assess vascular health. Finally, to link neuroimaging findings to cognitive functioning, enrolled CHD patients will undergo a neuropsychological test battery to investigate attention, executive functions, memory, visuospatial and sensorimotor domains which are typically altered in this population [11].

To summarize, we found a significantly higher burden of CMBs in adult ToF patients than in controls. WMHs volumetric burden was instead not significantly higher in patients, albeit a noteworthy association with NYHA class was observed. This proof-of-concept study is a pilot investigation inspirational for future researches, since the relationship between CHDs and cerebral aging has never been thoroughly investigated via neuroimaging tools. If confirmed, this finding could play a role for predicting and preventing transition to disability in patients with CHD such as ToF, who are currently able to reach older and older ages due to modern standard of care.

## Supporting information

S1 File Anonymized database used in this study. Detailed information on data used to obtain the results presented in this article.
(XLSX)
